# Integrated Comprehensive Care for Transcare and Gender Diverse Clinic Patients: An Assessment of Physical, Mental, and Social Needs

**DOI:** 10.7759/cureus.20544

**Published:** 2021-12-20

**Authors:** Kristy J Carlson, Jay A Irwin, Jayme R Dowdall, Sean C Figy, N. Jean Amoura

**Affiliations:** 1 Otolaryngology - Head and Neck Surgery, University of Nebraska Medical Center, Omaha, USA; 2 Sociology & Anthropology, University of Nebraska, Omaha, USA; 3 Plastic and Reconstructive Surgery, University of Nebraska Medical Center, Omaha, USA; 4 Obstetrics and Gynecology, University of Nebraska Medical Center, Omaha, USA

**Keywords:** healthcare inequality, healthcare services, healthcare facilities, healthcare, lgbtq, comprehensive care, transgender, gender diverse, transcare health clinic, gender-affirming

## Abstract

Introduction

There is a large body of research reporting the healthcare needs of groups identifying as lesbian, gay, bisexual, transgender, and/or queer (LGBTQ); however, a gap exists in the research literature because many epidemiological studies focus on sexual orientation rather than gender identify/incongruence. To address the lack of specific data from transgender and gender diverse (TGD) individuals, our organization designed and deployed a survey to assess the gender-affirming physical, mental, and social care needs of current patients.

Methods

A group of subspecialty physicians currently working with TGD patients created a list of questions and requested feedback from medical professionals familiar with the healthcare needs of this population. In addition, patients reviewed the survey for content and clarity. The final 68-item survey was distributed in April 2020 to patients or patients' representatives with an email address on file at the Nebraska Medicine Transgender Care Clinic (NMTCC). Participants were asked to respond to questions regarding their gender identity, their transition-related medical decisions, and their interest in services.

Results

Invitations were sent to 690 patients and 168 surveys were completed (response rate: 24.3%). Over 90% (n = 153) of the participants were patients and 9% (n = 15) answered survey questions on the patient’s behalf. A majority (77.2%) had started the medical transition (hormones or puberty blockers) in the past four years. Nearly half (46.4%) identified as trans women, 43.4% identified as trans men, and 10.2% indicated they were nonbinary or gender expansive. Participants' sex assigned at birth was 50.9% female and 46.1% male. Most patients (n = 149; 92%) reported currently receiving hormone treatment within the Nebraska Medicine healthcare system. Results indicated the highest level of clinical services interest was primary care (38.4%), gender-affirming surgery (73.5%), voice therapy (49.0%), and hair removal (37.5%). In addition, participants were very likely to participate in support groups with "people of similar gender identity" (32.9%), with "others around my age" (28.6%), and "including a mix of ages and identities" (26.9%).

Discussion

This study suggests that our TGD patients would utilize integrated services to access a variety of clinical and non-clinical services. Ongoing community engagement and direct feedback from patients are critical to the success and growth of our gender-affirming care clinic. The results of this study will inform the planning and further evolution of a program designed to build trust and address health inequities for TGD individuals throughout the region.

## Introduction

In the United States, the narrow and rigid societal definition of gender has expanded due to the scholarly work and efforts of lesbian, gay, bisexual, transgender, and queer (LGBTQ) advocates [1]. Although there is a large body of research regarding the healthcare needs of this broad LGBTQ population, gender identity/incongruence (transgender and queer) refers to personal experiences and individuality that are vastly different from sexual orientation (lesbian, gay, and bisexual) [2]. Transgender and gender diverse (TGD) people are often marginalized because their “health needs are frequently rendered invisible by a focus on sexual identities rather than on gender identities in health research” [3]. Further, it is difficult to identify health risks, outcomes, and disparities among the TGD population due to a lack of group data from US epidemiological studies [4].

Some efforts to document this lack of TGD-focused information are underway. A 2016 report published by the US Department of Health and Human Services outlined plans to focus specifically on the health and well-being of approximately 1.4 million transgender individuals [5,6]. In addition, Healthy People 2030 highlighted the need to understand this unique group by including a targeted national objective to “increase the number of national surveys that collect data on transgender populations” [7]. Although the medical field has made progress in gender-affirming treatment, research that contributes to the knowledge base specific to TGD individuals is necessary to inform integrated and patient-centered care [8].

One of the few sources of detailed information regarding this population is the 2015 U.S. Transgender Survey, which revealed higher levels of poverty, unemployment, psychological distress, suicide attempts, and discrimination in healthcare compared to the general population [9]. An update to this survey was delayed due to the coronavirus disease 2019 (COVID-19) pandemic [10]; however, recent studies have reinforced the initial findings. TGD populations consistently report difficulty accessing care [11,12] and significant disparities exist for TGD people receiving appropriate and consistent healthcare [13-15]. In light of the COVID-19 pandemic, TGD health and well-being have been negatively impacted due to increased isolation and delay in gender-affirming care as hospital resources were reallocated [16]. TGD populations regularly report increased violence and discrimination, particularly among trans people of color [17], including fears and experiences with discrimination in healthcare settings [18]. In the face of stigma and violence, substance use remains a health concern facing TGD populations [19,20]. These complex factors contribute to health disparities leading to negative outcomes among individuals in this high-risk group.

The current literature review reinforces the notion that integrating physical/mental health and social services may be critical to improving health outcomes for the TGD population [21]. Information specific to this population will contribute to the body of literature and assist in addressing health disparities among the TGD people. Gender-affirming care requires a team of healthcare providers to address clinical and non-clinical needs including primary and specialty care, mental health, pharmacy, and social services. The purpose of this study was to (1) design a survey to assess gender-affirming physical, mental, and social care needs; (2) distribute the survey to current Nebraska Medicine Transgender Care Clinic (NMTCC) patients; and (3) identify high-priority clinical and non-clinical services.

## Materials and methods

Nebraska Medicine Transgender Care Clinic

The NMTCC, a component of a university-based tertiary healthcare system, serves patients aged 8-84 years in a five-state region. Most are referred by personal contacts or support groups and seek gender-affirming hormone treatment. In August 2016, the location of NMTCC was moved from a women’s health clinic offering gender-affirming hormones to a dedicated specialty clinic. Current services offered on-site include obstetrics and gynecology, psychiatry, plastic surgery, and HIV care for all TGD individuals. Patients are referred to designated clinicians throughout the healthcare system (primary care, ENT, urology) for other gender-affirming care. In addition, a monthly support group for parents and caregivers of transgender youth is facilitated by a therapist at the specialty clinic.

Survey development

The survey items were created by a group of subspecialty physicians brainstorming potential services. These services included options that were being provided through the NMTCC as well as services that were known to be of interest to TGD populations. Draft survey questions were distributed to medical professionals within the organization. In addition, patients reviewed the survey and provided feedback on content and clarity. The survey was edited, finalized, and transferred to a secure, web-based application designed to support data capture for research studies. A copy of the survey is located in the Appendix.

Survey distribution

In April 2016, the NMTCC began collecting a list of patients seeking gender-affirming care. The 68-item anonymous survey was distributed in April 2020 to 707 participants with an email address on file in the electronic health records (EHR) database. A parent or guardian's address was recorded if the patient was a minor (<19 years of age, the age of majority in Nebraska). A total of 690 invitations were sent after excluding 17 that were “undeliverable.” Participants were asked to respond to questions regarding their gender identity, their transition-related medical decisions, and interest level in various services the NMTCC could provide to them in the future. Statistical Package for the Social Sciences (SPSS) version 27.0 (IBM Corp, Armonk, NY) was used for data analyses.

This study was initially completed as a quality improvement project. The IRB protocol (# 143-21-EX) was reviewed by the University of Nebraska Medical Center Office of Regulatory Affairs prior to submitting the manuscript for publication. Following the review of the IRB application, the principal investigator received the following response: "Per the information provided in the application where a quality improvement project was performed and now you plan to publish this information, the Office of Regulatory Affairs (ORA) determined this project does not constitute human subject research as defined at 45CFR46.102. Therefore, it is not subject to federal regulations. No further action is required."

## Results

A total of 168 surveys were completed (response rate: 24.3%). Over 90% (n = 153) of the participants were patients and 9% (n = 15) answered survey questions on the patient’s behalf. Almost one-quarter (n = 39; 24.1%) started their medical transition (hormones or puberty blockers) less than one year ago. Over half (n = 86; 53.1%) began the process in the past four years and 22.8% (n = 37) had been in the process of medical transition for five or more years. Nearly half (46.4%) identified as trans women, 43.4% identified as trans men, and 10.2% indicated they were nonbinary or gender expansive. Participants' sex assigned at birth was 50.9% (n = 84) female and 46.1% male (n = 76). One responded “neither” to this question and four (2.4%) chose not to answer. Additional demographics are listed in Table 1.

**Table 1:** Demographics of transgender care clinic patients. The total number and percentage of patients are categorized by age group, gender identity, and treatment goals. ^1 ^Twenty-two participants were representatives responding on behalf of the patient. ​​​​​​​^2 ^Nonbinary individuals have a gender identity that is not solely masculine or feminine.

	n	%
Patient age^1^		
≤18 years	22	13.4
19-39 years	95	57.9
≥40 years	47	28.6
Sex assigned at birth		
Female	85	51.5
Male	76	46.1
Choose not to answer	4	2.4
Gender Identity		
Trans Woman, woman, male-to-female	77	46.4
Trans man, man, female-to-male	72	43.4
Nonbinary^2^, gender expansive	17	10.2
Treatment goals		
Feminizing therapy	76	47.2
Masculinizing therapy	71	44.1
N/A, not interested in surgical procedures	14	8.7

Survey participants were asked about the location of their current treatment and if they were interested in receiving treatment specifically at the NMTCC. Most patients (n = 149; 92%) reported currently receiving hormone treatment within the current healthcare system. In addition to an interest in primary care services (n = 58; 38.4%), there was a desire for several other services. These included gender-affirming surgery (n = 114; 73.5%), voice therapy (n = 73; 49.0%), hair removal (n = 53; 37.5%), weight loss management (n = 54; 36.5%), mental health therapy (n = 51; 33.6%), psychiatry for prescribing mental health medications (n = 44; 30.1%), pharmacy (n = 37; 24.3%), and smoking cessation (n = 18; 12.4%). Almost half (n = 75; 46.9%) of the survey participants were interested in feminizing surgery/procedures and 44% (n = 71) were interested in masculinizing surgery/procedures. Only 8.8% (n = 14) reported no interest in surgery/procedures. A breakdown of specific types of procedures can be found in Table 2.

**Table 2:** Transgender patients' interest in feminizing or masculinizing surgical procedures. The total number and percentage of patients indicating an interest in feminizing and masculinizing procedures. The survey was used to gauge interest in a variety of current and future surgical services performed at Nebraska Medicine.

	Interested, n (%)	Not interested, n (%)
Feminizing procedures (n = 76)		
Breast augmentation	43 (56.6)	33 (43.4)
Removal of testes (orchiectomy)	40 (52.6)	36 (47.4)
Genital reconstruction (vaginoplasty, vulvoplasty)	52 (68.4)	24 (31.6)
Facial feminization surgery	52 (68.4)	24 (31.6)
Tracheal shave to reduce the visual appearance of Adam’s apple	28 (36.8)	48 (63.2)
Voice pitch alteration (laryngoplasty)	36 (47.4)	40 (52.6)
Masculinizing procedures (n = 71)		
Top surgery (chest reconstruction)	43 (60.6)	28 (39.4)
Removal of uterus and/or ovaries (hysterectomy/oophorectomy)	54 (76.1)	17 (23.9)
Creation of penis from enlarged clitoris (metoidioplasty, “meta”)	28 (39.4)	43 (60.6)
Creation of penis from the skin of thigh or forearm (phalloplasty)	26 (36.6)	45 (63.4)
Facial masculinization surgery	14 (19.7)	57 (80.3)

Survey participants were very likely to participate in support groups "with people of a similar gender identity" (n = 53; 32.9%), "with others around my age" (n = 28.6%), and "including a mix of ages and identities" (n = 43; 26.9%). Participants were very interested in educational topics focused on finding a surgeon (n = 90; 53.6%), preparing for surgery (n = 89; 55.6%), and formally changing their gender marker (n = 82; 51.3%). Additional group activities and education needs are listed in Table 3.

**Table 3:** Transgender patients' interest in education topics and group activities. The Nebraska Medicine Transgender Care Clinic is in a unique position to offer support to transgender and gender diverse individuals through group activities and education. These survey items were helpful to clinic leadership in prioritizing the needs of patients.

	Very likely to participate, n (%)	Might participate, n (%)	Probably would not participate, n (%)
Group activities			
Support group with people of similar gender identity	53 (32.9)	83 (51.6)	25 (15.5)
Support groups with others around my age	46 (28.6)	84 (52.2)	31 (19.3)
Support groups including a mix of ages and identities	43 (26.9)	77 (48.1)	40 (25.0)
Social gatherings with other patients of the clinic	39 (23.9)	84 (51.5)	40 (24.5)
Group voice coaching	31 (19.5)	49 (30.8)	79 (49.7)
Support group for partner/spouse	18 (11.6)	32 (20.6)	105 (67.7)
Support group for parents/guardians	17 (10.8)	32 (20.4)	108 (68.8)
Support group for siblings	13 (8.3)	29 (18.5)	115 (73.2)
	Very interested, n (%)	Somewhat interested,n (%)	Not interested, n (%)
Education topics			
How to find a surgeon	90 (56.3)	40 (25.6)	29 (18.1)
Preparing for surgery	89 (55.6)	38 (23.8)	33 (20.6)
How to change your gender marker	82 (51.3)	27 (16.9)	51 (31.9)
Navigating health insurance	73 (45.9)	47 (29.6)	39 (24.5)
How to change your name	71 (44.4)	23 (14.4)	66 (41.3)
Financial assistance	61 (37.9)	40 (24.8)	60 (37.3)
Work discrimination	47 (29.9)	43 (27.4)	67 (42.7)
Employment opportunities	48 (29.8)	37 (23.0)	76 (47.2)
Hair and makeup	48 (29.8)	26 (16.1)	87 (54.0)
Weight loss	44 (27.5)	35 (21.9)	81 (50.6)
Shopping for clothes	41 (25.5)	29 (18.0)	91 (56.5)
Sexual health and sexuality	39 (24.2)	54 (33.5)	68 (42.2)
Navigating schools (elementary, high school, college)	26 (16.5)	17 (10.8)	115 (72.8)
Fertility and family building	22 (14.0)	24 (15.3)	111 (70.7)
Help with housing, food, shelter	22 (13.6)	30 (18.5)	110 (67.9)
Binders and packers	20 (12.7)	30 (19.0)	108 (68.4)
Eating disorders/anorexia	14 (9.0)	27 (17.4)	114 (73.5)
Quitting smoking	14 (9.0)	9 (5.8)	133 (85.3)
Breast prostheses and articles for tucking	14 (8.8)	25 (15.6)	121 (75.6)
Child custody issues	10 (6.3)	7 (4.4)	141 (89.2)
Substance use	2 (1.3)	10 (6.5)	141 (92.2)

## Discussion

Ding et al. recommended centralization of care services for transition- and non-transition-related experiences [22] and a systematic review suggests utilization may increase with the integration of health and social services [21]. Our study reinforces this work regarding select healthcare needs specific to NMTCC patients responding to the survey (Appendix). Results reveal that TGD patients would utilize integrated services to access primary care, pharmacy, psychiatry/psychology, social work, legal counsel, otolaryngology, and plastic surgery. The highest level of interest was in feminizing/masculinizing procedures, finding a surgeon, preparing for surgery, changing a gender marker, and group activities to create a sense of community and social support. To our knowledge, this is the first needs assessment to identify physical, mental, and social needs specifically for the TGD subgroup within the LGBTQ population.

A healthcare program designed to address health inequities for transgender individuals fills an urgent need for this underserved population [23]. Indeed, Morenz et al. suggest components of transgender health programs should include case management, administrative support, primary care in addition to an inclusive environment, continuous quality improvement, and ongoing needs assessments [23]. In keeping with these recommendations, as the NMTCC grows and evolves, our goal is to continue patient-centered care informed by continued needs assessments. Within the past five years, we have noted high utilization of expanded services beyond gender-affirming hormones. As aspirational care models are tempered with the current realities in healthcare resourcing such as budgetary, space, and personnel concerns, it is our intention to clearly advocate for the needs of the patients we serve. In addition, we plan to reassess on an interval basis to see how well the articulated needs were addressed in a continuous quality improvement model. Traditionally, health research specific to the TGD population was limited to HIV/AIDS or mental health. Additional information is available for this group; however, results were extrapolated from epidemiological studies with a general focus on sexual orientation, omitting survey items regarding the gender-affirming needs of TGD individuals. Although there is an insufficient number of healthcare providers with specific competence in gender-affirmation services, transgender health care is shifting in response to growing social awareness and an appreciation of the biological components of gender identity [24].

An integrated care model for TGD patients involves strategies to create a supportive infrastructure and environment (e.g., signs, inclusive bathrooms, and patient education materials). Structurally, such a model will strive to integrate comprehensive care from the intake process through follow-up to treatment and/or procedures. Personnel that are trained specifically in the needs and considerations for gender-affirming care are fundamental to success, including a key team member who completes intake and assists patients in navigating the complex healthcare system. Pharmaceutical care that is available onsite increases access to hormone treatment, including guidance on coverage for costly prescriptions and injection education. In addition to hormone treatment, basic services, and preventive care, primary care providers fill a specific (and often unmet) need for TGD patients. These providers work with an established team of gender-affirming surgeons (i.e., urology, plastic surgery, and otolaryngology) to guide their transition through consultations and using centralized scheduling. This structural approach eliminates the typical barriers related to insurance and disjointed referrals that contribute to negative health outcomes.

Research reports that increased access to gender-affirming care is associated with improved mental health [11,21]. Psychiatric and psychological care is also needed to improve and maintain overall health. Incorporating these services directly into an integrated model eliminates interactions with practitioners who are unfamiliar with the specific issues that impact members of the TGD community [9]. Other specializations offered through this model include phlebotomy and medical social work to facilitate psychosocial resource referrals and navigate insurance preauthorization requests and surgical consults.

The results of the survey provide valuable information to the NMTCC leadership; however, there are limitations. Our data included only current patients responding to the survey and is not representative of all NMTCC patients, or the broader TGD community. Although the results may not be applied to other TGD populations, this survey may be easily replicated. Further, operationalizing the findings is not possible due to our narrow focus on defining comprehensive care needs for patients seeking gender-affirming care. Future research needs to continue to investigate this patient population and expand participation by TGD individuals who may not have financial or practical access to health care.

## Conclusions

In summary, we found patients desire access to clinical and non-clinical services. There was high interest in surgery/procedures, legal assistance, and group activities to create a sense of community and social support. In a cycle of continuous quality improvement, we will solicit feedback from patients to build trust, increase utilization of resources, and contribute to positive health outcomes. Ongoing engagement with community groups and patients is critical to innovation and success as we work within the constraints of the healthcare system.

## Appendices

**Table 4:** Transgender care clinic needs assessment survey.

Please respond to the following questions.					
1.	Are you our patient, or are you completing this survey for a family member or friend who is our patient?				
	I am the patient	__			
	I am completing this for the patient	__			
2.	Age of patient:				
	<19	__			
	19-29	__			
	30-39	__			
	40-49	__			
	50-59	__			
	60+	__			
3.	When did you start medical transition (hormones or puberty blockers)?				
	<1 year ago	__			
	1-2 years ago	__			
	3-4 years ago	__			
	5+ years ago	__			
4.	What sex was assigned at birth?				
	Female	__			
	Male	__			
	Neither	__			
	Choose not to answer	__			
5.	What is your gender identity?				
	What services would you like to receive at the transgender clinic?	Currently receiving or have received in the past	Currently receiving or have received in the past elsewhere	Would like to receive at the transgender clinic	Not interested
6.	Hormone treatment	___	___	___	___
7.	Primary care (family medicine, general medicine, pediatrics)	___	___	___	___
8.	Pharmacy (picking up your medication)	___	___	___	___
9.	Voice therapy	___	___	___	___
10.	Psychiatry (prescribing mental health medications)	___	___	___	___
11.	Therapy (mental health, psychology)	___	___	___	___
12.	Gender-affirming surgery	___	___	___	___
13.	Hair removal (laser or electrolysis)	___	___	___	___
14.	Weight loss management	___	___	___	___
15.	Help with quitting smoking	___	___	___	___
16.	Please list other services you are interested in?				
17.	What type of surgery are you interested in, if any?				
	Feminizing procedures	__			
	Masculinizing procedures	__			
	N/A, not interested in surgical procedures	__			
	If feminizing procedures are selected: which procedures are you interested in? (check all that apply)				
18.	Breast augmentation	__			
19.	Removal of testes (orchiectomy)	__			
20.	Genital reconstruction (vaginoplasty, vulvoplasty)	__			
21.	Genital reconstruction (vaginoplasty, vulvoplasty)	__			
22.	Facial feminization surgery (FFS)	__			
23.	Tracheal shave to reduce the visual appearance of Adam's apple	__			
24.	Voice pitch alteration (laryngoplasty)	__			
	If masculinizing procedures are selected: which procedures are you interested in? (check all that apply)				
25.	Top surgery (chest reconstruction)	__			
26.	Removal of uterus and/or ovaries (hysterectomy/oophorectomy)	__			
27.	Creation of penis from enlarged clitoris (metoidioplasty, "meta")	__			
28.	Creation of penis from the skin of thigh or forearm (phalloplasty)	__			
29.	Facial masculinization surgery	__			
30.	If you are interested in any other operations, please list here:				
	What additional services are you interested in?	Very likely to participate	Might participate	Probably would not participate	
31.	Social gatherings with other patients of the clinic	___	___	___	
32.	Support groups with others around my age	___	___	___	
33.	Support group with people of similar gender identity	___	___	___	
34.	Support group including a mix of ages and identities	___	___	___	
35.	Support group for parents/guardians	___	___	___	
36.	Support group for partner/spouse	___	___	___	
37.	Support group for siblings	___	___	___	
38.	Group voice coaching	___	___	___	
	What HEALTH information topics are you interested in?	Very interested	Somewhat interested	Not interested	
39.	How to find a surgeon	___	___	___	
40.	Preparing for surgery	___	___	___	
41.	Fertility and family building options	___	___	___	
42.	Sexual health and sexuality	___	___	___	
43.	Weight loss	___	___	___	
44.	Eating disorders/anorexia	___	___	___	
45.	Quitting smoking	___	___	___	
46.	Substance use	___	___	___	
	What LEGAL information topics are you interested in?	Very interested	Somewhat interested	Not interested	
47.	How to change your name	___	___	___	
48.	How to change your gender maker on documents	___	___	___	
49.	Work discrimination	___	___	___	
50.	Child custody issues	___	___	___	
51.	Navigating schools (elementary, high school, college)	___	___	___	
	What LIFE ESSENTIALS information topics are you interested in?	Very interested	Somewhat interested	Not interested	
52.	Financial assistance	___	___	___	
53.	Navigating health	___	___	___	
54.	Employment opportunities	___	___	___	
55.	Help with housing, food, shelter	___	___	___	
	What PERSONAL APPEARANCE information topics are you interested in?	Very interested	Somewhat interested	Not interested	
56.	Hair and makeup	___	___	___	
57.	Shopping for clothes	___	___	___	
58.	Binders and packers	___	___	___	
59.	Breast prostheses and articles for tucking	___	___	___	
60.	If you are interested in more information on topics not listed on this or the last few pages, what would you like to see or participate in?				
	How are you interested in getting information on these topics?	Very interested	Possibly interested	Not interested	
61.	Group workshops in person	___	___	___	
62.	Members-only teleconference (such as Zoom meeting)	___	___	___	
63.	Facebook live event	___	___	___	
64.	Newsletter	___	___	___	
65.	If interested in a different format, please indicate:				
66.	Any additional comments or suggestions are welcome:				
